# Cost Attributable to Nosocomial Bacteremia. Analysis According to Microorganism and Antimicrobial Sensitivity in a University Hospital in Barcelona

**DOI:** 10.1371/journal.pone.0153076

**Published:** 2016-04-07

**Authors:** Marta Riu, Pietro Chiarello, Roser Terradas, Maria Sala, Enric Garcia-Alzorriz, Xavier Castells, Santiago Grau, Francesc Cots

**Affiliations:** 1 IMIM (Hospital del Mar Medical Research Institute), Barcelona, Spain; 2 Universitat Autònoma de Barcelona (UAB), Barcelona, Spain; 3 School of Nursing, Hospital del Mar, Barcelona, Spain; 4 Department of Epidemiology and Evaluation, Hospital del Mar, Barcelona, Spain; 5 Redissec (Red de Investigación en Servicios Sanitarios en enfermedades crónicas), Madrid, Spain; 6 Department of Pharmacy, Hospital del Mar, Barcelona, Spain; University College London, UNITED KINGDOM

## Abstract

**Aim:**

To calculate the incremental cost of nosocomial bacteremia caused by the most common organisms, classified by their antimicrobial susceptibility.

**Methods:**

We selected patients who developed nosocomial bacteremia caused by *Staphylococcus aureus*, *Escherichia coli*, *Klebsiella pneumoniae*, or *Pseudomonas aeruginosa*. These microorganisms were analyzed because of their high prevalence and they frequently present multidrug resistance. A control group consisted of patients classified within the same all-patient refined-diagnosis related group without bacteremia. Our hospital has an established cost accounting system (full-costing) that uses activity-based criteria to analyze cost distribution. A logistic regression model was fitted to estimate the probability of developing bacteremia for each admission (propensity score) and was used for propensity score matching adjustment. Subsequently, the propensity score was included in an econometric model to adjust the incremental cost of patients who developed bacteremia, as well as differences in this cost, depending on whether the microorganism was multidrug-resistant or multidrug-sensitive.

**Results:**

A total of 571 admissions with bacteremia matched the inclusion criteria and 82,022 were included in the control group. The mean cost was € 25,891 for admissions with bacteremia and € 6,750 for those without bacteremia. The mean incremental cost was estimated at € 15,151 (CI, € 11,570 to € 18,733). Multidrug-resistant *P*. *aeruginosa* bacteremia had the highest mean incremental cost, € 44,709 (CI, € 34,559 to € 54,859). Antimicrobial-susceptible *E*. *coli* nosocomial bacteremia had the lowest mean incremental cost, € 10,481 (CI, € 8,752 to € 12,210). Despite their lower cost, episodes of antimicrobial-susceptible *E*. *coli* nosocomial bacteremia had a major impact due to their high frequency.

**Conclusions:**

Adjustment of hospital cost according to the organism causing bacteremia and antibiotic sensitivity could improve prevention strategies and allow their prioritization according to their overall impact and costs. Infection reduction is a strategy to reduce resistance.

## Introduction

Bacteremia is a severe infection with high mortality rates. Nosocomial bacteremia constitutes an important part of healthcare related infections. In a study conducted in 12 Spanish hospitals that aimed to evaluate the costs of adverse events, healthcare infections were the group with highest incremental costs, representing 64.2% of the total cost of episodes with adverse events [1]. Nosocomial bacteremia increases length of stay, hospital costs and mortality rates, as it can trigger more serious events such as septic shock, occasionally with multiple organ failure, and death. The incidence rate of nosocomial bacteremia has been estimated to be between 113 and 189/100,000 inhabitants per year, with an associated mortality of between 21 and 38/100,000 per year [2]. The additional per-patient cost at hospital level has been estimated at € 14,735 [3] or € 12,853 [4]. In a systematic review that analyzed variations in analytical methods to estimate the incremental costs of nosocomial infections, the results for nosocomial bacteremia varied from $ 5,875 to $ 86,500 [5]; this disparity reflects differences in patient characteristics, the type of infection, the causative microorganisms, and the calculation methods.

Bacteremia caused by multidrug-resistant microorganisms has a worse prognosis than that is caused by antimicrobial-susceptible microorganisms, because multidrug resistance delays and reduces the choice of appropriate treatment. Consequently, adverse effects could be more frequent and the selected antimicrobials are usually more expensive and, due to their broader spectrum, could promote the development of new antimicrobial resistances [6–8]. Population-based studies have estimated the additional costs of antimicrobial resistance at $ 20 billion in the USA [9] and 1.5 billion annually in Europe, of which more than 900 million are direct hospital costs [10]. In the case of bacteremia, the excess cost of multidrug resistance has been estimated at $16,918 per patient [11]. Unfortunately, the prevalence of multidrug resistant microorganisms is constantly growing, despite various strategies for prevention, which is a concern for health professionals [9,10].

The excess cost associated with nosocomial bacteremia is avoidable and can be used as a measure of the impact of these infections. However, some authors have suggested that traditional methods overestimate the incremental cost due to the presence of various types of bias. First, the possibility of developing nosocomial bacteremia is due, in part, to length of exposure. Consequently, a longer hospital stay increases the risk of acquiring an infection. In addition, a hospital-acquired infection increases the length of stay, converting the length of stay into a variable directly related to a time-dependent bias [12–14]. Further biases can also be related to the lack of complete information on all confounding variables (default variable bias) or if the sample analyzed does not include adequate control cases (selection bias) [13–15].

The main source of cost is hospital analytical cost accounting, which includes the cost of all activities conducted in the process of providing healthcare to hospitalized patients and all relevant cost typologies [14,16].

Due to the difficulty of identifying the multitude of activities performed in a hospital, currently few hospitals have developed detailed per patient cost accounting. Consequently, most studies analyzing bacteremia costs are based on secondary estimates rather than real costs [5, 17,18]. These methodologies lose sensitivity in showing the variability of costs due to the specific circumstances that affect the type and number of activities performed in the treatment of each patient according to their characteristics and needs. The use of costs based on analytical cost accounting systems ensures that the variability of clinical practice is sufficiently captured in terms of resource use. A recently published study on the economic impact of adverse events showed the advantages and strengths of the use of activity-based costing and full costing techniques in healthcare economic evaluation [1].

Differentiating the incremental cost of bacteremia by microorganisms according to their antimicrobial sensitivity or resistance would contribute to a better understanding of the magnitude of the economic burden of resistance. In addition, such an analysis would help to evaluate the effectiveness of initiatives attempting to antimicrobial resistance and would provide valuable information on the areas that should be prioritized to control healthcare infections [19–22].

### Objective

The main aim of the study was to calculate the incremental cost of nosocomial bacteremia caused by the most common organisms, classified by antibiotic sensitivity.

## Material and Methods

A retrospective study was conducted at Hospital del Mar, Barcelona, a teaching hospital equipped with 400 beds that provides medical and surgical care and has a catchment area of 300,000 inhabitants.

The study population consisted of patients admitted to the hospital from 2005 to 2012. All discharges were classified with the all-patient refined-diagnosis-related group (APR-DRG) v. 24.0 grouper. Admitted patients who developed nosocomial bacteremia caused by *Staphylococcus aureus*, *Escherichia coli*, *Klebsiella pneumoniae* or *Pseudomonas aeruginosa* and all control patients classified within the same APR-DRG groups were selected. These microorganisms were analyzed because of their high prevalence, and, in our environment, often have multidrug resistance.

Nosocomial bacteremia was defined according to the Center for Disease Control definitions [23]. Magiorakos[24] criteria were used to identify multidrug resistant microorganisms (Table 1).

**Table 1:** Criteria used to identify multidrug-resistant microorganisms.

Microorganisms	Antimicrobial category	Antimicrobial agents
	Aminoglycosides	Amikacin
		Gentamicin
		Tobramycin
	Carbapenems	Ertapenem
*E*. coli*		Imipenem
*K*. pneumoniae*		Meropenem
	1st or 2nd generation cephalosporins	Cefazoline
		Cefuroxime
	3rd or 4th generation cephalosporins	Cefotaxime
		Ceftazidime
		Cefepime
	Aminoglycosides	Amikacin
		Gentamicin
		Tobramycin
	Monobactams	Aztreonam
	Cephalosporins with antipseudomonal activity	Ceftazidime
*P*. aeruginosa*		Cefepime
	Quinolones	Ciprofloxacin
		Levofloxacin
	Ureidopenicillins	Piperacillin + Tazobactam
	Carbapenems	Imipenem
		Meropenem
	Polymyxins	Colistin
	Penicillins	Oxacillin
*S*. aureus**		Cloxacillin
		Methicillin
	Glycopeptides	Vancomycin

* Microorganisms were considered to be multidrug-resistant if identified as such by the microbiologist or if they had developed resistance to three or more antibiotics family.

** Microorganisms were considered to be multidrug-resistant if they developed resistance to one antibiotic family.

Admissions grouped into non-specific APR-DRG were excluded. Patients with two or more episodes of bacteremia during the same hospital stay were also excluded because of their specific risk factors [25].

The dependent variable was the cost of the hospitalization episode. The main explanatory variable was the presence of nosocomial bacteremia. Additional study variables were age and sex, type of admission (emergency or elective), type of treatment (medical or surgical), discharge status (alive or deceased), admission diagnosis, the presence of complications, level of comorbidities as measured by the Elixhauser index [1], intensive care unit (ICU) admission, per-patient cost previous to the bacteremia as a weighted measure of length of hospital stay prior to bacteremia detection and the cost weight of the adjacent APR-DRG group.

The total costs of hospital admissions with nosocomial bacteremia caused by the selected microorganisms were analyzed by taking into account the antimicrobial susceptibility of each microorganism. This group of patients was compared with the control group.

Clinical information on the hospitalization process of each patient included in the study was obtained from the minimum dataset of hospital discharges and from the hospital information system, which has an established cost accounting systems (full-costing) in which cost distribution is based on activity-based criteria[26]. All costs were adjusted to 2012 price levels according to the national price index published by Spain’s National Institute of Statistics [27]. Admissions with bacteremia were identified using the dataset collected by the infection control department, which prospectively follows up all episodes of bacteremia.

A descriptive analysis was performed of all variables of admissions with and without bacteremia and of microorganism sensitivity. The Chi-square test was used to compare categorical variables and the comparison of mean values was performed using ANOVA test.

A generalized linear model with binomial distribution and logit function was fitted to estimate, for each admission, the probability of developing bacteremia (propensity score) and was used for propensity score matching adjustment [14,28]. The model was specified the variables previously described with logarithmic transformations for the APR-DRG cost weight and for cost previous to the bacteremia. Subsequently, the propensity score was included in a generalized linear models with the Gamma distribution and the log link function to adjust the incremental cost of patients who developed bacteremia, as well as differences in this cost, depending on whether the microorganism was multidrug-resistant or multidrug-sensitive. The econometric model included the same variables used for the estimation of the propensity score (see S1 File for details).

The study was approved by the Ethics Committee for Clinical Research of Hospital del Mar, Parc de Salut Mar. Any patient-level data was anonymized, and no additional informed consent was required.

## Results

There were 177,378 discharges in the study period, of which 0.96% had an episode of bacteremia (n = 1,703). Of these, 669 (39.3%) were episodes of nosocomial bacteremia caused by the above-mentioned microorganisms. Finally, 571 admissions with bacteremia met the inclusion criteria and 82,022 treated in the same period and grouped in the same APR-DRG who did not develop nosocomial bacteremia were included in the control group. Table 2 shows the characteristics of the selected episodes. At hospital admission, patients from both groups had similar levels of comorbidities (Elixhauser index) and requirements for surgical intervention, although patients who developed bacteremia were more likely to be admitted to the ICU and had higher mortality and resource consumption (measured by APR-DRG weight and average cost).

**Table 2:** Patients without and with bacteremia, characteristics, and average cost of the episode.

	Patients		Patients with bacteremia				
	without bacteremia		multidrug-sensitive		multidrug-resistent		P-value
Number of patients	82,022		404		167		
Male sex (N, %)	43,256	52.7	249	61.6	101	60.5	<0.000
Age (mean, SD)	67.1	17.0	64.7	15.8	67.2	14.7	0.017
Urgent admission (N, %)	53,171	64.8	291	72.0	126	75.4	<0.000
Surgical patient (N, %)	34,534	42.1	193	47.8	67	40.1	0.062
Intensive care unit admission (N, %)	4,508	5.5	128	31.7	51	30.5	<0.000
Exitus (N, %)	3,852	4.7	112	27.7	52	31.1	<0.000
Elixhauser index (mean, SD)	2.6	1.9	2.6	1.7	2.8	1.9	0.428
APR-DRG weight (mean, SD)	1.1539	0.8405	1.9435	2.2947	1.8310	2.0532	<0.000
Length of hospital stay (mean, SD)	10.8	10.3	32.2	28.0	37.2	24.4	<0.000
Length of hospital stay prior to bacteremia (mean, SD)	-		14.5	15.2	18.5	13.4	-
Total cost observed (mean, SD)	6,750	7,619	24,960	31,154	28,143	23,964	<0.000

Abbreviation: APR-DRG = all-patient refined-diagnosis related group.

Among the 571 episodes with bacteremia, the most frequently isolated microorganism was *E*. *coli* (184 episodes), followed by *S*. *aureus* (108 episodes). The least frequent microorganism was *P*. *aeruginosa* (86 episodes), which nevertheless showed the highest percentage (40.7%) of episodes with multidrug resistance. Overall, 29.2% of microorganisms showed multidrug resistance (Table 3). About 16% of nosocomial bacteremia were polymicrobial.

**Table 3:** Distribution of bacteremia according to the causative organism and its antibiotic sensitivity.

	Multidrug-sensitive		Multidrug-resistant		
	N	%	N	%	Total
*S*. *aureus*	70	64.8	38	35.2	108
*E*. *coli*	145	78.8	39	21.2	184
*K*. *pneumoniae*	69	67.6	33	32.4	102
*P*. *aeruginosa*	51	59.3	35	40.7	86
Polimicrobial	69	75.8	22	24.2	91
Total	404	70.8	167	29.2	571

The total cost was € 568,464,250. The 571 admissions with bacteremia represented 0.69% of analyzed admissions and 2.6% of total observed cost (Table 4).

**Table 4:** Average and total cost among all patients according to the causative organism and its antibiotic sensitivity.

			Observed cost	
Patients	N	%	mean	total
Without bacteremia	82,022	99.3	6,75	553,680,489
With bacteremia	571	0.69	25,891	14,783,761
Total	82,593	100.0	6,883	568,464,250
**Microorganism group**				
*S*. *aureus* (MDSM)	70	12.3	22,851	1,599,541
*S*. *aureus* (MDRM)	38	6.7	24,468	929,792
*E*. *coli* (MDSM)	145	25.4	21,883	3,173,003
*E*.*coli* (MDRM)	39	6.8	16,754	653,392
*K*. *pneumoniae* (MDSM)	69	12.1	25,222	1,740,292
*K*. *pneumoniae* (MDRM)	33	5.8	35,100	1,158,312
*P*. *aeruginosa* (MDSM)	51	8.9	30,201	1,540,266
*P*. *aeruginosa* (MDRM)	35	6.1	38,872	1,360,505
Polymicrobial (MDSM)	69	12.1	29,431	2,030,733
Polymicrobial (MDRM)	22	3.9	27,179	597,938
Total number of patients with bacteremia	571	100.0	25,891	14,783,775

MDSM = multidrug-sensitive microorganism; MDRM = multidrug-resistant microorganism.

The mean cost of admissions with bacteremia was € 25,891. Nosocomial bacteremia caused by multidrug-resistant *P*. *aeruginosa* was the most costly with a mean observed cost of € 38,872; episodes caused by multidrug-resistant *E*. *coli* had the lowest mean cost, € 16,754 (Table 4). The mean incremental cost was estimated at € 15,151 (CI, € 11,570 to € 18,733) and was related to an increase of € 8,651,221 in total hospital costs. As shown in Table 5, multidrug-resistant *P*. *aeruginosa* bacteremia had the highest incremental cost, € 44,709 (CI € 34,559 to € 54,859). Despite being one of the less common microorganisms, *P*. *aeruginosa* had a substantial impact on total costs, amounting to € 1,564,817. The mean incremental cost was € 8,872 for multidrug-resistant *E*. *coli* bacteremia and was € 10,481 (CI € 8,752 to € 12,210) for antimicrobial-susceptible *E*. *coli* nosocomial bacteremia, which represented the second highest total cost (€ 1,519,735). Despite its lower mean incremental cost compared with other causative agents, antimicrobial-susceptible *E*. *coli* nosocomial bacteria had a major impact due to its high frequency.

**Table 5:** Average incremental cost and impact on cost of the distinct causative organisms and their antibiotic sensitivity.

		Patients	Incremental cost			Impact on costs	
Microorganism group	N	%	Mean	Confidence interval		Total	%
*S aureus (MSSA)*	70	12,3	9.350	7.215	11.485	654.500	7,6
*MRSA*	38	6,7	14.372	10.426	18.317	546.136	6,3
*E coli* (MDSM)	145	25,4	10.481	8.752	12.210	1.519.745	17,6
*E coli* (MDRM)	39	6,8	8.872	6.588	11.155	346.008	4
*K pneumoniae* (MDSM)	69	12,1	13.864	11.019	16.709	956.616	11,1
*K pneumoniae* (MDRM)	33	5,8	18.208	12.663	23.754	600.864	6,9
*P aeruginosa* (MDSM)	51	8,9	16.042	11.956	20.127	818.142	9,5
*P aeruginosa* (MDRM)	35	6,1	44.709	34.559	54.859	1.564.815	18,1
Polymicrobian (MDSM)	69	12,1	19.009	14.917	23.100	1.311.621	15,2
Polymicrobian (MDRM)	22	3,9	15.138	6.932	23.344	333.036	3,8
Total	571	100	15.151	11.570	18.733	8.651.221	100

MDSM = multidrug-sensitive microorganism; MDRM = multidrug-resistant microorganism.

Multidrug-sensitive nosocomial polymicrobial bacteremia also had a major impact on cost, amounting to € 19,009 (CI € 14,917 to € 23,100 IC), and represented a total incremental cost of € 1,311,987.

## Discussion

The 571 admissions developing nosocomial bacteremia during the 8 years analyzed increased total hospital costs by € 8,651,221, more than € 1,000,000 per year. The mean additional cost was € 15,151 per admission. Substantial differences were found between organisms. Multidrug-resistant *P*. *aeruginosa* bacteremia had the highest economic impact, with a mean incremental cost of € 44,709.

The characteristics and implications of the *S*. *aureus* bacterium have been constantly studied since the first epidemic of methicillin-resistant *S*. *aureus* (MRSA) in the 1960s [29]. The observed costs related to multidrug-sensitive *S aureus* (MSSA) ranged from $ 9,839 to $ 59,245. Reported costs for MRSA have ranged from $ 11,045 to $ 84,436 [30,31]. A study analyzing patients with *S*. *aureus* bacteremia in 27 Spanish hospitals [31] reported that the cost of MSSA was € 9,839 and the cost of MRSA was € 11,044, slightly lower than those found in the present work.

Two previous studies have evaluated the cost of MRSA bacteremia compared with MSSA bacteremia using propensity score matching techniques, although their findings are not clearly comparable with those of this study. The first [32] included community-acquired bacteremia in selected cases; the estimated cost was $ 8,355 (MSSA) vs. $ 9,369 (MRSA). The second [33] analyzed the cost of patients with and without ICU admission separately; among the former, those who developed MRSA bacteremia were associated with higher costs (with an odds ratio of 2 relative to MSSA), while non-ICU patients showed no significant difference in cost.

*E*. *coli* was the most frequent bacteremia found in this study (32.2% of total episodes) and showed the lowest multidrug resistance (21.2%). Its mean incremental cost estimate was lower than those of other microorganisms but it had the second highest total economic impact due to its high frequency. The adjusted cost of multidrug-resistant *E*. *coli* was lower than that for multidrug-sensitive episodes.

*E*. *coli* is one of the most frequent microorganisms causing bacteremia, whether hospital- or community-acquired [34–36], and has an increasing share of multidrug resistance [13, 36]. Nevertheless, very few studies have analyzed the impact of this microorganism on hospital cost. This could be because *E*. *coli* bacteremia usually results from urinary tract infections, which are generally low-severity and produce few complications. However, because of its frequency, this microorganism warrants study.

In a systematic review of the economic impact of multidrug resistance [18], only one of the studies described the costs of *E*. *coli* bacteremia and found observed mean costs of € 13,709 for multidrug-resistant microorganism, € 8,683 for multi-sensitive microorganism and € 5,026 for those related to multidrug resistance; these costs are lower than those observed in our cohort with this organism, which can be explained by the inclusion of community-acquired bacteremia episodes.

Other studies mentioned in the same review analyzed the costs of infections caused by *E*. *coli* and *K*. *pneumoniae* and included different types of infections, not only bacteremia. In our study, the mean observed cost was € 21,883 for *E*. *coli* multidrug-sensitive episodes, € 16,754 for multidrug-resistant episodes, € 25,222 for multidrug-sensitive *K*. *pneumoniae* episodes and € 35,100 for multidrug-resistant episodes. The results were considerably higher for *K*. *pneumoniae* episodes; consequently, although both microorganisms belong to the same family, their inclusion in the same study does not seem an appropriate strategy. The cost described in the above-mentioned review and attributable to multidrug resistance for both microorganisms ranged from $ 1,587 to $ 30,093. The authors attribute this substantial disparity to the differences in cost of living among the countries included in the study. We believe that this may explain some of the differences but that differences in the patients enrolled and in calculation methods may also have had an influence.

An analysis of the cost of infections (except urinary tract infections) caused by *E*. *coli* and *K*. *pneumoniae* also found high incremental costs, with a mean cost of $ 41,353 for multidrug-resistant and $ 24,902 for multidrug-sensitive microorganisms [37]. Likewise, in patients with multidrug-resistant *E*. *coli* or *K*. *pneumoniae* nosocomial infection, Roberts et al [8] found a mean cost of $ 39,403, similar to our results for bacteremia caused by multidrug-resistant *K pneumoniae* (€ 35,100) but clearly higher than the mean cost of bacteremia caused by multidrug-resistant *E*. *coli* (€ 16,754).

*P*. *aeruginosa* was the microorganism with the highest proportion of multidrug resistance and also showed a higher estimated incremental cost for both multidrug-sensitive episodes (€ 16,042) and multidrug-resistant episodes (€ 44,709). These results are consistent with those found by our team in a previous study, in which the cost of infection/colonization by *P*. *aeruginosa* was € 4,933 for multidrug-sensitive episodes and € 12,351 for multidrug-resistant episodes, despite the lower costs in our study; however, in that study, bacteremia as well as infection and colonization were included in the cost analysis [22]. Findings from other studies show higher costs in patients infected with or colonized by *P*. *aeruginosa*. For episodes with imipenem-resistance the mean hospital post-culture cost was $ 251,495 for multidrug-resistant microorganisms and was $ 166,196 for multidrug-sensitive microorganisms [21]. Neidell et al [38] found an incremental cost of $ 25,300 for infection with multidrug-resistant *P*. *aeruginosa* compared to multidrug-sensitive *P aeruginosa*. These results are very similar to the findings of the present study, since the estimated cost of nosocomial episodes of multidrug-resistant *P*. *aeruginosa* bacteremia was € 28,667 higher than that of multidrug-sensitive *P*. *aeruginosa* bacteremia. Nevertheless, the high levels of cost found in these analyses may be because most infections caused by *P*. *aeruginosa* occur in advanced stages of illness or in immunocompromised patients [21].

There is evidence that a significant proportion of nosocomial infections could be avoided. Findings from a review article suggest that the figure could be higher than 20% [39]. Some authors believe that the ability of experts to properly compare the effectiveness of prevention strategies for nosocomial infections is severely limited by the multiplicity of pathogens and the changing epidemiology of multidrug resistance in different centers [40]. As clearly shown in literature, several strategies to prevent nosocomial infections and reduce antibiotic resistance have been implemented [41–44]. Nevertheless, most of the reported experiences have been conducted in strongly controlled environments, such as ICUs, where, in the USA [45], a 58% reduction in nosocomial catheter-related bacteremia was estimated in 2008 compared with 2004. In addition, after application of specific hospital programs, two studies found decreases of 41.7% [42] and 73.9% [43] in bacteremia associated with central venous catheters.

The success of clinical measures in reducing antimicrobial resistance has varied [9,10, 19] and some measures have had substantial success, such as in Denmark, where the rate of MRSA bacteremia decreased from 32% in 1968 to less than 2% in the last few years [29].

This study analyzed the hospital costs of all nosocomial bacteremia caused by the most common organisms in a tertiary-care teaching hospital in Barcelona. The aim was to expand the findings of other studies that included only a single microorganism in the analysis and which were often based on a single outbreak of bacteremia or on bacteremia in a single hospital area. The analytical structure of our study has limitations: first, we used information from a single hospital center, which could affect extrapolation of the results. Second, we excluded health care-associated bacteremia, which is always the result of a previous contact with the hospital. Thus, the episode of hospitalization represents an entirely avoidable cost. Finally, costs were calculated from the perspective of the hospital and inpatient care.

The strength of this study lies in the exhaustiveness and thoroughness of the process of identifying bacteremia. Episodes were selected according to the protocols of the professionals involved in the infection control program and not by using administrative databases, as often occur in similar studies. It has been estimated that administrative-only information identifies only 20% of infections [46]. Importantly, this study analyzed real hospital costs elaborated with an activity-based costing system instead of tariffs or estimates from other sources.

In line with the results of other authors [12,13], our results showed that length of hospital stay is an important factor that should always be taken into account and controlled for. Admissions with bacteremia, on average, developed the infection on the 14th day of stay while admissions without bacteremia had a mean length of stay of 10 days; in other words, admissions without bacteremia are discharged before the date when bacteremia usually develops. In this analysis we used a propensity-score matching approach to minimize potential time-dependent bias and selection bias.

This study also highlights the importance of conducting interventions to control multidrug resistance, as conducted in some hospitals by implementing specific programs [47].

The adjustment of hospital cost for the organism causing bacteremia and for antibiotic sensitivity allows prevention strategies to be improved and prioritized according to their global impact and costs. Importantly, infection reduction is a strategy to reduce antimicrobial resistance.

## Supporting Information

S1 File Supporting information. (DOCX)
