# Supplementary material for: Cost Attributable to Nosocomial Bacteremia. Analysis According to Microorganism and Antimicrobial Sensitivity in a University Hospital in Barcelona
Source: PLoS One. 2016 Apr 7;11(4):e0153076. doi: 10.1371/journal.pone.0153076 (PMC4824502; doi:10.1371/journal.pone.0153076)
Supplement: S1 File — (DOCX) [file pone.0153076.s001.docx]

**Supporting information.**

| Table 1. GLM (binomial family and logit link) model for the estimation of propensity score. Dependent variables is episode of bacteremia. | | | | | | |
| --- | --- | --- | --- | --- | --- | --- |
|  |  |  |  |  |  |  |
|  | Coef. | Std. Err. | z | P>z | [95% Conf. | Interval] |
|  |  |  |  |  |  |  |
| Lenght of stay | 0.0727474 | 0.0025866 | 28.12 | 0 | 0.0676777 | 0.0778171 |
| Log (Cost previous to bacteremia) | -1.495.091 | 0.0693931 | -21.55 | 0 | -1.631.098 | -1.359.083 |
| Age | -0.0053662 | 0.0028081 | -1.91 | 0.056 | -0.01087 | 0.0001377 |
| Log (APR-DRG adjacent grup cost weight) | 0.6110629 | 0.0955102 | 6.4 | 0 | 0.4238663 | 0.7982595 |
| Discharge type (1=Surgical) | 0.1869841 | 0.131955 | 1.42 | 0.156 | -0.071643 | 0.4456113 |
| Elixhauser index | -0.0264021 | 0.0274932 | -0.96 | 0.337 | -0.0802879 | 0.0274836 |
| Gender (1=Female) | -0.2223457 | 0.095288 | -2.33 | 0.02 | -0.4091068 | -0.0355846 |
| Emergency (1=yes) | -0.2847335 | 0.1131221 | -2.52 | 0.012 | -0.5064488 | -0.0630183 |
| Deceased (1=yes) | 1.803.327 | 0.1136873 | 15.86 | 0 | 1.580.504 | 202.615 |
| ICU (1=yes) | 2.096.147 | 0.1395959 | 15.02 | 0 | 1.822.544 | 236.975 |
| Constant | 6.673.143 | 0.5754832 | 11.6 | 0 | 5.545.217 | 7.801.069 |

| Figure 1. Bias reduction among explanatory variables after propensity score matching. | | | | |
| --- | --- | --- | --- | --- |
|    \|  \| \| --- \| |  |  |  |  |
|  |  |  |  |  |
|  |  |  |  |  |
|  |  |  |  |  |
|  |  |  |  |  |
|  |  |  |  |  |
|  |  |  |  |  |
|  |  |  |  |  |
|  |  |  |  |  |
|  |  |  |  |  |
|  |  |  |  |  |
|  |  |  |  |  |
|  |  |  |  |  |
|  |  |  |  |  |
|  |  |  |  |  |

| Table 2. GLM (gamma family and log link) model for the estimation of incremental cost of bacteremia. Dependent variables is total cost of hospital discharge. | | | | | | |
| --- | --- | --- | --- | --- | --- | --- |
|  |  |  |  |  |  |  |
|  | Coef. | Std. Err. | z | P>z | [95% Conf. | Interval] |
|  |  |  |  |  |  |  |
| Propensity score | -0.0539539 | 0.0080695 | -6.69 | 0 | -0.0697698 | -0.038138 |
|  |  |  |  |  |  |  |
| S. aureus (MDRM) | 0.8919012 | 0.0094878 | 94.01 | 0 | 0.8733054 | 0.9104969 |
| E.coli (MDRM) | 0.7713801 | 0.0076564 | 100.75 | 0 | 0.7563738 | 0.7863865 |
| K. pneumoniae (MDRM) | 0.8927974 | 0.0145224 | 61.48 | 0 | 0.864334 | 0.9212608 |
| P. aeruginosa (MDRM) | 1.442.514 | 0.0083744 | 172.25 | 0 | 1.426.101 | 1.458.928 |
| Polymicrobial (MDRM) | 0.7897293 | 0.0096478 | 81.86 | 0 | 0.77082 | 0.8086386 |
|  |  |  |  |  |  |  |
| S. aureus (MDSM) | 0.705339 | 0.0072356 | 97.48 | 0 | 0.6911576 | 0.7195205 |
| E. coli (MDSM) | 0.7296313 | 0.0063007 | 115.8 | 0 | 0.7172821 | 0.7419805 |
| K. pneumoniae (MDSM) | 0.7465029 | 0.007995 | 93.37 | 0 | 0.730833 | 0.7621729 |
| P. aeruginosa (MDSM) | 0.7222882 | 0.0077437 | 93.27 | 0 | 0.7071108 | 0.7374655 |
| Polymicrobial (MDSM) | 0.872104 | 0.0081147 | 107.47 | 0 | 0.8561995 | 0.8880085 |
|  |  |  |  |  |  |  |
| Ref: Year=2005 |  |  |  |  |  |  |
| Year=2006 | 0.1069333 | 0.0076546 | 13.97 | 0 | 0.0919306 | 0.121936 |
| Year=2007 | 0.0118463 | 0.0078769 | 1.5 | 0.133 | -0.003592 | 0.0272847 |
| Year=2008 | 0.1330442 | 0.0070849 | 18.78 | 0 | 0.119158 | 0.1469305 |
| Year=2009 | 0.3679328 | 0.0082628 | 44.53 | 0 | 0.351738 | 0.3841275 |
| Year=2010 | 0.2818447 | 0.0077753 | 36.25 | 0 | 0.2666054 | 0.297084 |
| Year=2011 | 0.2384732 | 0.0084933 | 28.08 | 0 | 0.2218266 | 0.2551197 |
| Year=2012 | 0.0697365 | 0.0083219 | 8.38 | 0 | 0.0534259 | 0.0860471 |
| Age | -0.0023103 | 0.0001297 | -17.81 | 0 | -0.0025645 | -0.002056 |
| Log (APR-DRG adjacent grup cost weight) | 0.4108536 | 0.004405 | 93.27 | 0 | 0.40222 | 0.4194871 |
| Discharge type (1=Surgical) | 0.1050146 | 0.0062234 | 16.87 | 0 | 0.0928169 | 0.1172123 |
| Elixhauser index | 0.0165835 | 0.0011705 | 14.17 | 0 | 0.0142894 | 0.0188777 |
| Gender (1=Female) | 0.0465518 | 0.0043993 | 10.58 | 0 | 0.0379294 | 0.0551743 |
| Emergency (1=yes) | -0.0128363 | 0.0050273 | -2.55 | 0.011 | -0.0226897 | -0.002983 |
| Deceased (1=yes) | 0.2616002 | 0.0157022 | 16.66 | 0 | 0.2308245 | 0.2923759 |
| ICU (1=yes) | 0.9370616 | 0.0193675 | 48.38 | 0 | 0.899102 | 0.9750211 |
| Constant | 8.243.056 | 0.047883 | 172.15 | 0 | 8.149.207 | 8.336.905 |
